# Five-year cumulative incidence and progression of age-related macular degeneration: results from the German population-based Gutenberg Health Study (GHS)

**DOI:** 10.1007/s00417-021-05312-y

**Published:** 2021-08-23

**Authors:** Christina A. Korb, Hisham Elbaz, Alexander K. Schuster, Stefan Nickels, Katharina A. Ponto, Andreas Schulz, Philipp S. Wild, Thomas Münzel, Manfred E. Beutel, Irene Schmidtmann, Karl J. Lackner, Tunde Peto, Norbert Pfeiffer

**Affiliations:** 1 Department of Ophthalmology, University Medical Center of the Johannes Gutenberg-University Mainz, Mainz, Germany; 2 Department of Ophthalmology, Otto-von-Guericke University Magdeburg, Magdeburg, Germany; 3 Center for Thrombosis and Hemostasis (CTH), University Medical Center of the Johannes Gutenberg-University Mainz, Mainz, Germany; 4 Preventive Cardiology and Preventive Medicine, Center for Cardiology, University Medical Center of the Johannes Gutenberg-University Mainz, Mainz, Germany; 5 German Center for Cardiovascular Research (DZHK), Partner Site Rhine-Main, Mainz, Germany; 6 Center for Cardiology, Cardiology I, University Medical Center of the Johannes Gutenberg-University Mainz, Mainz, Germany; 7 Department of Psychosomatic Medicine and Psychotherapy, University Medical Center of the Johannes Gutenberg-University Mainz, Mainz, Germany; 8 Institute for Medical Biostatistics, Epidemiology and Informatics, University Medical Center of the Johannes Gutenberg-University Mainz, Mainz, Germany; 9 Institute of Clinical Chemistry and Laboratory Medicine, University Medical Center of the Johannes Gutenberg-University Mainz, Mainz, Germany; 10 NIHR Biomedical Research Centre at Moorfields Eye Hospital NHS Foundation Trust and UCL Institute of Ophthalmology, London, UK; 11 Centre for Public Health, Queen’s University Belfast, Belfast, Northern Ireland UK

**Keywords:** Age-related macular degeneration, Cumulative incidence, Progression, Gutenberg Health Study

## Abstract

**Purpose:**

Age-related macular degeneration (AMD) is a major cause of visual impairment and blindness. This study evaluates the incidence and progression of AMD in a large German cohort.

**Methods:**

The Gutenberg Health Study (GHS) is a population-based, prospective, observational cohort study in Germany that includes 15,010 participants between 35 and 74 years of age. The baseline examination, including fundus photography, was conducted between 2007 and 2012, and the 5-year follow-up examination was performed between 2012 and 2017. AMD grading of fundus photographs was performed according to the Rotterdam Eye Study classification. The 5-year cumulative incidence and progression of AMD were calculated. Poisson regression analysis was conducted to investigate factors associated with the cumulative incidence and progression of AMD.

**Results:**

Six-thousand-eight-hundred-eighty-eight participants (49.8%, n = 3427 female) were included in the analysis. AMD prevalence was 8.5% [95% CI: 7.9–9.2%] at baseline and 10.3% [95% CI: 9.6–11.1%] at follow-up. The cumulative 5-year-incidence was 2.0% [1.7–2.4%]. AMD progression within 5 years was seen in 18.1% [95% CI: 15.1–21.5%] of the participants. AMD incidence and AMD progression were associated with higher age, for each 10-year increase in age, the risk of AMD doubles (RR = 2.30), and the risk of progression of the disease is increased by 1.6. while AMD incidence also with pseudophakic status.

**Conclusions:**

In summary, this population-based sample provides substantial epidemiologic data from a large German cohort, including data on progression and cumulative incidence of macular degeneration in younger age groups. AMD progression over 5 years is common in the German population, 18.1% of subjects with AMD showed progression in at least one eye in this time frame and is associated with higher age. Nevertheless, although usually defined to occur over the age of 50, in this cohort AMD occurred in 0.5% and AMD progression occurred in 5.4% of those already affected in the youngest age group before 50 years of age.

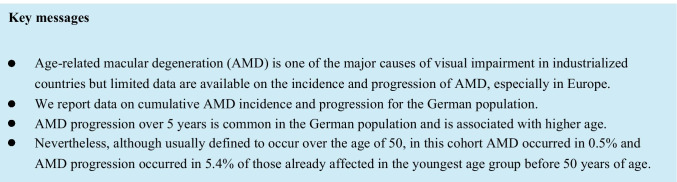

## Introduction

Age-related macular degeneration (AMD) is one of the major causes of visual impairment in industrialized countries [1]. With the elderly population steadily growing, the burden of disease related to the impairment of visual function will increase in the future [2]. The earliest signs of AMD are drusen underneath the retinal pigment epithelium (RPE) and regions of altered retinal pigment epithelium with hyper- and hypopigmentation [1]. The advanced stages of this disease are responsible for the most severe visual loss and can be categorized into dry (geographic atrophy) and wet forms (choroidal neovascularization [CNV] or exudative AMD) [1].

In the population-based Blue Mountains Eye Study, 3654 participants 49 years of age and older were examined; the 15-year cumulative incidence was 22.7% for early AMD and 6.8% for late AMD, indicating that higher age is a strong risk factor for both early and late AMD [3]. The Beaver Dam Eye Study, a population-based study conducted in the USA, included 3917 persons 43 to 86 years of age at the time of baseline examination and found 15-year cumulative incidences of 14.3% for early AMD and 3.1% for late AMD [4]. In Europe, few population-based prospective studies have been conducted and differed in diagnostic parameters and study design. The Rotterdam Eye Study in the Netherlands and the Coimbra Eye Study in Portugal assessed the 6.5 year cumulative incidence of early and late AMD, while the AGES-R study in Iceland, the POLA and the ALIENOR studies in France and the Copenhagen City Eye Study included a 5-, 3- 4-year and 14-year follow-up, respectively [5–10].

Several risk factors for AMD have been identified [11]. Well-studied examples are genetic factors [12, 13], alcohol intake [14], smoking [15], skin sensitivity to sun and iris color [16]. Additionally, associations between AMD and arterial hypertension, cardiovascular disease, cerebrovascular disease, dyslipidemia, chronic kidney disease and neurodegenerative disorders have been reported [17]. Obesity is related to the risk of AMD [18]; furthermore, metabolic syndrome, obesity, high glucose levels, and high triglyceride levels were predictors of progression to late AMD [19]. Limited data are available on the incidence and progression of AMD, especially in Europe. Therefore, the aim of this study is to determine the 5-year cumulative incidence and progression of AMD in the Gutenberg Health Study (GHS), a German population-based cohort, and to evaluate associations with established and potential risk factors.

## Materials and methods

### Study population

The GHS is a population-based, prospective, single-center cohort study conducted at the medical center of the Johannes Gutenberg University Mainz in Germany [20]. The sample was randomly drawn via local residents’ registration offices and equally stratified by sex for each decade of age. The exclusion criteria for participation in the GHS were physical or mental inability or insufficient knowledge of German to participate in the examinations at the study center. The baseline examination, which included a total of 15,010 participants 35 to 74 years of age, was conducted from 2007 to 2012 and consisted of an ophthalmological examination and several general and cardiovascular examinations as well as interviews and questionnaires. The ophthalmic portion of the examination has been described in detail elsewhere [21]. In brief, we measured objective refraction, distance-corrected visual acuity, and intraocular pressure and conducted visual field testing, pachy- and keratometry, and posterior segment photography. The follow-up examinations were conducted between 2012 and 2017. Overall, 12,423 (82.8%) subjects of the GHS cohort underwent follow-up examination after 5 years. We included all participants who had gradable images from both baseline and 5-year follow-up available. The GHS is divided into three similar recruiting waves, each of which is representative of the study population, to allow subsample analyses.

### AMD grading

The fundus images were taken with a nonmydriatic fundus camera (Visucam PRO NMTM, Carl Zeiss AG, Jena, Germany) in a darkened room and with the pupil at its natural width. Three photographs were taken of each eye: one each at 30° and 45° centered on the optic nerve, and one at 30° centered on the macula. The fundus images were assessed by a German board-certified consultant of ophthalmology (HEB) using the Irfan View 4.41 program and a 24″ high-resolution monitor. In case of ambiguity, the grading was discussed with a senior grader from the reading center of Moorfields Eye Hospital (TP).

The definitions for AMD lesions closely followed the Rotterdam Eye Study classification as previously applied to GHS data by Korb et al. [22]. Lesions in the macular region were categorized as early and late maculopathy. Early AMD included stages 1a, 1b, 2a, 2b, and 3. Late AMD comprised neovascular and atrophic changes (stages 4a and 4b). Stage 5, which is maculopathy not related to AMD, included lesions that are considered to be the result of non-AMD-related disease and was excluded from the AMD grading (Table 1). Small, hard drusen were excluded from the AMD grading according to the classification of the International AMD Epidemiology Study Group [23].

**Table 1:** Classification of the stages of age-related macular degeneration (AMD) in the Gutenberg Health Study

AMD category	Stage/category	Explanation
No AMD	0a	No signs of AMD
No AMD	0b	Hard drusen (< 63 μm, ≤ 10) only
No AMD	0c	Hard drusen (< 63 μm, > 10) only
Early AMD	1a	Soft, distinct drusen (≥ 63 μm) only
Early AMD	1b	Pigmentary abnormalities only, no soft drusen (≥ 63 μm)
Early AMD	2a	Soft, indistinct drusen (≥ 125 μm) or reticular drusen only
Early AMD	2b	Soft, distinct drusen (≥ 63 μm) with pigmentary abnormalities
Early AMD	3	Soft, indistinct (≥ 125 μm) or reticular drusen with pigmentary abnormalities
Late AMD	4a	Atrophic AMD
Late AMD	4b	Neovascular AMD
Excluded from analysis	5	Maculopathy unrelated to AMD
Excluded from analysis	6a	Not gradable (due to photo quality)
Excluded from analysis	6b	Not gradable (due to obscuring lesion)
Excluded from analysis	6c	Not gradable (due to missing photo)

For AMD incidence and progression, the follow-up images were graded in direct comparison to the baseline images. The definition of incidence of AMD in this study is person-based and is based on the development of new lesion(s) in at least 1 eye at the 5-year follow-up among those not featuring the same stage of the lesion noted at baseline. In case in which different stages of AMD were noted in the right and left eye, the classification for the more advanced lesion was taken, while in case of multiple lesions, the proximity to the fovea was judged as the most important factor, followed by the criterion “most advanced” lesion [24].

### Ocular characteristics

The ophthalmic examination has been described in detail by Höhn et al. [21]. Refraction and distance-corrected visual acuity were measured using a Humphrey Automated Refractor/Keratometer (HARK) 599 (Carl Zeiss AG, Jena, Germany) in both eyes, beginning with the right eye. Best-corrected visual acuity was measured using built-in Snellen charts ranging from 20/400 to 40/20 (decimal 0.05 to 2.0). Below that visual acuity, we used a visual acuity chart at a distance of one meter up to 20/800, and then counting fingers, hand movements, and testing of light perception. The spherical equivalent was calculated as the spherical correction value plus half the cylindrical power. Intraocular pressure was measured with an air-puff noncontact tonometer (Nidek NT-2000; Nidek, Co., Gamagori, Japan), again beginning with the right eye. The mean of three measurements within a 3-mmHg range was obtained for each eye. History of eye disease was assessed in a short interview preceding the eye examination.

### Risk factors and comorbidities

Family history as a proxy for genetic risk was assessed with the questionnaire item “Do you know of any AMD cases among your parents or siblings?” Diabetes mellitus was defined by fulfilling one of the following criteria: diabetes mellitus diagnosed by a physician, known therapy (oral medication or insulin), or glycated hemoglobin (HbA1c) ≥ 5%. Dyslipidemia was defined by a low-density lipoprotein (LDL) to high-density lipoprotein (HDL) ratio (LDL/HDL) of > 3.5, triglyceride levels after overnight fasting > 150 mg/dL, lipid-lowering medication, or diagnosis by a physician. Hypertension was defined by the use of antihypertensive medication, systolic blood pressure > 140 mm Hg, diastolic blood pressure > 90 mm Hg, or diagnosis by a physician Self-reported smoking was dichotomized into current smoking and nonsmoking (including former smoking). Obesity was defined as BMI >  = 30 m^2^/kg. Cardiovascular disease was defined as history of ischemic heart disease, myocardial infarction, stroke, or peripheral arterial disease.

### Sociodemographic characteristics

Socioeconomic status (SES) was based on income, education and occupation according to the German Health Update 2009 (GEDA) with a possible range of 3 to 21 (3 indicates the lowest SES, and 21 indicates the highest SES) [25].

### Statistical analysis

The data management team performed quality controls for all data and checked for completeness and correctness using predefined algorithms and quality plausibility controls. Analyses were performed using GNU R (Version 3.3.1) [26]. For descriptive analyses, we calculated the mean of the spherical equivalent and the intraocular pressure of both eyes for every participant. To assess potential nonresponder bias, we compared the characteristics of participants who had a complete data set with those of participants with missing information.

Five-year cumulative incidence was calculated based on the dataset of subjects who showed no signs of AMD at baseline. Of those, subjects who displayed any signs of AMD at the 5-year follow-up examination were defined as incident cases, and those without signs of AMD were defined as nonincident cases. Potential risk factors were evaluated using robust Poisson regression analysis. The following parameters were included as independent variables: sex, age, smoking, alcohol consumption, BMI, HbA1c, LDL, HDL, triglycerides, mean arterial blood pressure, spherical equivalent, pseudophakia at baseline, and family history of AMD. If necessary, variables were transformed.

Progression of AMD was computed based on the dataset of subjects who showed any signs of AMD at baseline. Progression was defined as being classified as at least one AMD category higher at the 5-year follow-up examination than at the baseline examination. Potential risk factors were evaluated using robust Poisson regression analysis with the same covariates as described above.

## Results

Of the GHS population, 6888 participants (49.8%, n = 3427 female) had images of sufficient quality available from both the baseline and the follow-up examinations and were included in the analysis. The median age was 52.9 years (standard deviation 10.5 years) at baseline, and 4.5% (n = 313) of the participants had a family history of AMD. A detailed description of the sample characteristics is provided in Table 2.

**Table 2:** Characteristics of the study sample (n = 6.888) of the German population-based Gutenberg Health Study (GHS) at baseline

	Men (3461)	Women (3427)
Mean (± SD) age at baseline [years]	53.0 ± 10.6	52.8 ± 10.5
Mean (± SD) socioeconomic status	14.20 ± 4.49	12.66 ± 4.14
Eye characteristics		
Median (iQR) Visual acuity [logMAR]	0 (0/0.10)	0 (0/0.10)
Mean (± SD) IOP [mmHg]	14.24 ± 2.87	14.02 ± 2.59
Median (iQR) spherical equivalent [dpt]	− 0.25 (− 1.38/0.56)	− 0.12 (− 1.31/0.75)
Mean (± SD) CCT [µm]	555 ± 35	551 ± 35
Family history of AMD, % (n)	3.5% (122)	5.6% (191)
History of eye surgery, % (n)	4.9% (171)	6.1% (209)
Pseudophakia, % (n)	2.3% (80)	3.3% (112)
AMD (Baseline) (yes), % (n)	9,0% (313)	7.8% (268)
Stage 1, % (n)	6.8% (234)	4.8% (165)
Stage 2, % (n)	1.4% (48)	1.6% (56)
Stage 3, % (n)	0.4% (15)	1.2% (40)
Stage 4, % (n)	0.5% (16)	0.2% (7)
Cardiovascular risk factors		
Obesity (yes), % (n)	23.9% (827)	22.3% (763)
Diabetes (yes), % (n)	7.8% (271)	4.7% (162)
Smoking (yes), % (n)	19.3% (666)	18.0% (616)
Arterial hypertension (yes), % (n)	50.1% (1734)	39.9% (1368)
Dyslipidemia (yes), % (n)	52.1% (1802)	30.1% (1030)
Alcohol consumption (yes, > 10 g/d for women and > 20 g/d for men), % (n)	29.4% (1018)	24.0% (822)

*IQR*, interquartile range; *SD*, standard deviation; *logMAR*, logarithm of the minimum angle of resolution

Nonresponder analysis comparing participants with gradable images from both baseline and follow-up to participants without gradable images at both time points is presented in Table 3. This analysis (Table 3) revealed that nonresponders have a higher prevalence of pseudophakia and a higher spherical equivalent compared to participants with images from both the baseline and the follow-up visit but do not differ with respect to cardiovascular risk factors.

**Table 3:** Nonresponder analysis comparing participants with gradable images from both the baseline and the follow-up visit to participants without images at both time points (weighted for age and sex)

Weighted for age and sex	Responder (6888)	Nonresponder (8122)
Sex (women)	49.8%	49.2%
Mean age [years]	52.9 ± 10.5	56.8 ± 11.3
Smoking (yes)	18.6%	23.2%
Alcohol consumption (yes (> 10 g/d for women; > 20 g/d for men))	26.7%	25.7%
BMI [kg/m^2^] Median (iQR)	26.3 (23.7/29.6)	26.6 (23.8/30.1)
HbA1c [%] Median (iQR)	5.5 (5.2/5.7)	5.5 (5.2/5.8)
LDL [mg/dl] Mean (± SD)	139 ± 35	138 ± 36
HDL [mg/dl] Mean (± SD)	57.7 ± 15.7	56.8 ± 15.6
Triglycerides [mg/dl] Mean (± SD)	102.4 (76.0/142.0)	105.0 (77.0/149.0)
Mean arterial blood pressure [mmHg]	98.4 ± 11.0	98.5 ± 11.2
Spherical equivalent [dpt]	− 0.19 (− 1.375/0.625)	− 0.125 (− 1.25/0.75)
Pseudophakia (yes)	2.8%	4.0%
Family history of AMD (yes)	4.5%	4.5%

*iQR*, interquartile range; *SD*, standard deviation; *dpt*, diopter; *BMI*, body mass index; *HbA1c*, Glycosylated Hemoglobin, Type A1C; *LDL*, Low-density lipoprotein cholesterol; *HDL*, High-density lipoprotein cholesterol

The AMD prevalence was 8.5% [7.9–9.2%] at baseline and 10.3% [9.6–11.1%] at follow-up, evaluated by cross-sectional analysis. The cumulative 5-year-incidence was 2.0% [1.7–2.4%], and the cumulative 5-year-progression was 18.1% [15.1–21.5%]. The number of events and their frequencies by decade of age and sex are shown in Table 4. In this cohort, AMD occurred in 0.5% [0.3–1.0] and AMD progression took place in 5.4% [1.4–15.8] of the youngest age group before 50 years of age.

**Table 4:** Cumulative 5-year incidence and progression of age-related macular degeneration (AMD), stratified by sex and decade of age at follow-up, in the Gutenberg Health Study sample

		Frequency in % (95% confidence interval)			Number of events		
	Age decade	Men	Women	Total	Men	Women	Total
AMD incidence	**40–49 years**	0.4 [0.1; 1.1]	0.7 [0.3; 1.5]	0.5 [0.3; 1.0]	3	6	9
	**50–59 years**	1.3 [0.7; 2.2]	0.7 [0.3; 1.5]	1.0 [0.6; 1.6]	13	7	20
	**60–69 years**	2.0 [1.2; 3.3]	3.1 [2.0; 4.5]	2.5 [1.9; 3.5]	16	25	41
	**70–79 years**	6.3 [4.4; 8.9]	5.8 [3.9; 8.6]	6.1 [4.7; 7.9]	31	26	57
AMD progression	**40–49 years**	3.1 [0.2; 18.0]	8.3 [1.5; 28.5]	5.4 [1.4; 15.8]	1	2	3
	**50–59 years**	12.5 [6.2; 22.9]	9.4 [2.5; 26.2]	11.5 [6.4; 19.7]	9	3	12
	**60–69 years**	11.5 [6.1; 20.0]	20.9 [13.3; 30.9]	16.0 [11.2; 22.3]	11	19	30
	**70–79 years**	26.5 [18.9; 35.8]	22.3 [15.5; 31.0]	24.4 [19.1; 30.5]	30	27	57

Each 10-year increase in age is linked to a RR = 2.30 for 5-year cumulative AMD incidence. The risk of progression of the disease is increased by RR = 1.6 for each 10-year increase in age. Having had cataract surgery with intraocular lens implantation is associated with a 2.5-fold increase in risk for AMD incidence (p = 0.0019). A higher body-mass index was linked with higher risk for AMD incidence (RR = 1.05; p = 0.02), while the other evaluated factors were not associated with either incidence or progression of AMD. Details of the full models are shown in Table 5.

**Table 5:** Associations with cumulative 5-year incidence and progression of age-related macular degeneration (AMD) in the Gutenberg Health Study estimated using robust Poisson regression; baseline parameters were included

	Cumulative 5-year incidence of AMD			Cumulative 5-year progression of AMD		
	RR	95% CI	*p*	RR	95% CI	*p*
Sex (women)	0.91	0.60; 1.38	0.66	0.90	0.59; 1.39	0.63
Age [10 y]	2.30	1.83; 2.80	< 0.0001	1.60	1.22; 2.12	< 0.001
Smoking	1.36	0.84; 2.20	0.21	0.95	0.53; 1.69	0.86
Alcohol consumption (> 10 g/d for women and > 20 g/d for men)	1.07	0.73; 1.58	0.72	0.84	0.56; 1.27	0.42
BMI [kg/m^2^]	1.05	1.01; 1.09	0.017	1.02	0.97; 1.07	0.37
HbA1c [%]	1.09	0.83; 1.44	0.54	1.24	0.99; 1.56	0.06
LDL [mg/dl]	1.00	1.00; 1.01	0.62	1.00	1.00; 1.01	0.79
HDL [mg/dl]	1.01	0.99; 1.02	0.32	1.01	0.99; 1.02	0.34
Log (triglycerides [mg/dl])	0.89	0.53; 1.51	0.67	0.82	0.50; 1.33	0.42
Mean arterial blood pressure [mmHg]	0.99	0.97; 1.01	0.20	1.00	0.99; 1.02	0.67
Spherical equivalent [dpt]	0.97	0.90; 1.05	0.48	1.07	0.99; 1.16	0.11
Pseudophakia	2.52	1.41; 4.53	0.0019	0.86	0.42; 1.77	0.67
Family history of AMD	1.58	0.81; 3.09	0.18	1.10	0.57; 2.14	0.77

*AMD*, age-related macular degeneration; *RR*, risk ratio; *95% CI*, 95% confidence interval

## Discussion

Our study reports the incidence of AMD and AMD progression in a German cohort. The cumulative incidence over 5 years was 2.0%, and 18.1% of subjects with AMD showed progression in at least one eye in this time frame.

In the population-based Blue Mountains Eye Study in Australia, the 15-year cumulative incidence of early AMD was 22.7%, whereas it was 6.8% for late AMD [3]. In the USA, the Beaver Dam Eye Study found a 15-year cumulative incidence of 14.3% for early AMD and 3.1% for late AMD [4]. In the Blue Mountains Eye Study, persons aged 49 years and older were evaluated, in the Beaver Dam Eye Study, persons 43 to 86 years of age at the time of baseline examination were included [3, 4]. In the present study, the cumulative incidence over 5 years was 2.0% which is likely to be attributable to the younger age groups, as we included participants aged 35–74 at baseline. In Iceland, the Reykjavik Eye study analyzed the 5-year cumulative incidence of age-related maculopathy (ARM) and reported incidences of 1.1% at age 50–59 years, 6.7% at age 60–69 years and 12.8% at age 70–79 years [7]. These proportions are higher than those found in our study, in which 5-year cumulative incidences of 1.0%, 2.5% and 5.9% were observed in the respective age cohorts. Other population-based studies, including the Blue Mountain Eye Study, the Beaver Dam Eye Study and the Rotterdam Eye Study, reported comparable age dependencies [3, 4, 27]. The Copenhagen City Eye Study reported 14-year incidences of early and late ARM of 31.5% and 14.8%, respectively [10]. They evaluated an elderly population aged 60 to 80 years compared to our relatively young cohort aged 35 to 75 years [10]. Farhinha et al. described the 6.5-year incidence and progression of age-related macular degeneration in Portugal and found 6.5-year cumulative incidences of 10.7%, for early and 0.8% for late AMD, Progression occurred in 17.2% of patients aged 55 to 84 years [9]. In this cohort, we report AMD in individuals also under the age of 50, which is usually considered the lower age limit of age-related macular degeneration. Nevertheless, AMD occurred in 0.5% and AMD progression occurred in 5.4% of the youngest age group before 50 years of age.

Obesity is related to the risk of AMD, and body weight shows a dose-dependent association with AMD incidence, especially of the late stage of the disease, indicating that maintaining normal body weight and avoiding further weight gain may confer potential protection against this disease [18]. A 32% increase in the risk of developing late AMD was noted among obese individuals, while obesity showed no significant association with early AMD [18]. In the same study, elevated body mass index (BMI) showed a linear dose–response relationship to AMD risk in overweight and obese subjects, with a 2% increase for each 1 kg/m^2^ in BMI. Furthermore, metabolic syndrome, obesity, high glucose levels, and high triglyceride levels were predictors of progression to late AMD [19]. In a study investigating the role of statins in the development and progression of AMD, individuals with elevated lipid levels and those who had used statins for more than one year showed an increased risk of exudative AMD [28]. Nevertheless, evidence from currently available studies provides insufficient evidence for the conclusion that statins have a role in preventing or delaying the onset or progression of AMD [29].

On the other hand, a pooled analyses of these three studies showed that current smoking is associated with the incidence of late AMD (geographic atrophy or neovascular AMD), while other systemic factors such as body mass index, cholesterol level, blood pressure and diabetes were not associated. Consistent with this, we did not identify these factors as risk factors for cumulative AMD incidence or AMD progression.

We found that having had cataract surgery with intraocular lens implantation prior to our baseline examination was linked to higher cumulative AMD incidence. There are several ways to explain this link: first, inflammation induced by the surgery may lead to alterations within the retina and in the long term to the occurrence of AMD. A possible mechanism might also be photic retinal injury during the surgery or higher exposure to short-wavelength light in pseudophakic eyes, resulting in dry AMD due to the formation of free oxygen radicals [34, 31]. Second, having had cataract surgery may indicate that the eye has undergone age-related changes, including the development of cataracts, that may manifest in AMD later on as well; this may have affected our results, although our analysis was corrected for age. Nevertheless, the Beijing Eye Study reported that AMD occurrence was not associated with cataract or pseudophakia [32], and it remains unclear whether this association might differ according to ethnicity. For instance, the Los Angeles Latino Eye Study reported an association of prior cataract surgery with advanced AMD, increased retinal pigment and retinal pigment epithelial depigmentation [33]. Data from the Rotterdam Study showed that history of cataract surgery was associated with increased incidence of dry AMD, especially in homozygous CFH Y402H carriers [30]. Klein et al. found in the Beaver Dam Eye Study over a 20-year interval that cataract surgery was associated with increased incidence of late AMD but not of early AMD [34]. A recent systematic review also supports an association of cataract surgery with AMD [35]. Third, the altered configuration of the anterior chamber following cataract surgery may lead to induced posterior vitreous detachment, which may have an impact on AMD occurrence. A meta-analysis previously showed that posterior vitreous detachment is less frequent in AMD patients than in controls (neovascular AMD 0.77 (95% CI: 0.64–0.93), dry AMD 0.56 (95% CI: 0.27–1.14); consistent with this, we did not find pseudophakia to be a risk factor for AMD progression [36]. On the other hand, a recent meta-analysis reported that Asians are more likely to have AMD progression after cataract surgery, possibly due to differences between Asian and White patients in the genetic architecture of AMD [37, 38].

Although not detected in our study, a recent meta-analysis showed an increase in early AMD incidence of 1.06 per diopter increase in spherical equivalent [39]. Similar findings have been reported for prevalent AMD cases [40–42]. Two others studies found no significant association between myopia or hyperopia and the incidence of late or early AMD [43, 44].

The strengths of our study are the assessment of a very large cohort, the standardized study design and quality control, the broad assessment of phenotype information, and the use of population-based sampling. Despite the strengths, our study has several limitations. First, participants might be aware of an increased risk of AMD progression, as approximately one third of the population is in regular ophthalmic check-ups [45]. This might lead to changes in lifestyle factors such as smoking and nutrition. Second, there was a relevant proportion of ungradable images. We therefore performed a stepwise nonresponder analysis and did not find relevant differences in study participation except for age and sex. Therefore, we reported our descriptive statistics stratified for age and sex and age- and sex-weighted for the German population. In addition, image quality is an issue that might confound the estimation of the effect of pseudophakia, as pseudophakia leads to better image quality. We therefore adjusted our statistical model for image quality at baseline and follow-up and did not find relevant changes in the estimates. Taken together, residual confounding seems unlikely to explain our effect estimates. Third, we did not perform refraction measurements under cycloplegic conditions. Although in the described age range accommodation is limited, this might have influenced our results. Fourth, the recruiting was limited to participants below the age of 75 years at baseline; therefore, the decades with high AMD prevalence were not included.

In summary, we report data on cumulative AMD incidence and progression for the German population. We found that AMD occurred in 2.0% of the population and that 18.1% of subjects with AMD showed progression within 5 years. There is a strong age dependency, with higher risk in older subjects. Nevertheless, although usually defined as occurring over the age of 50, in this cohort AMD occurred in 0.5% and AMD progression occurred in 5.4% of the youngest age group before 50 years of age.

## Data Availability

The analysis presents clinical data of a large-scale population-based cohort with ongoing follow-up examinations. This project constitutes a major scientific effort in which high methodological standards and detailed guidelines for analysis and publication are applied to ensure that scientific analysis is conducted at the highest level. Therefore, the data are not made available to the scientific community outside the established and controlled workflows and algorithms. To meet the need for the verification and reproducibility of our scientific findings, we offer access to data at the local database in accordance with the ethics vote upon request at any time. The GHS steering committee, which comprises a member of each involved department and the head of the Gutenberg Health Study (PSW), convenes once each month. The steering committee makes decisions regarding internal and external access by researchers and use of the data and biomaterials based on research proposals supplied by the researchers. Interested researchers should submit their requests to the head of the Gutenberg Health Study (Philipp S. Wild; philipp.wild@unimedizin-mainz.de). More detailed contact information is available at the homepages of the GHS (www.gutenberghealthstudy.org) and the ophthalmic branch of the GHS (www.unimedizin-mainz.de/augenklinik/forschung/gutenberg-gesundheitsstudie.html).

## Abbreviations

*AMD*: Age-related macular degeneration
*ARM*: Age-related maculopathy
*BMI*: Body mass index
*CI*: Confidence interval
*GHS*: Gutenberg Health Study
*RR*: Risk ratio
*SES*: Socioeconomic status
